# First-Principles Investigation of the Effects of *B*-Type Medium Entropy Local Sublattice on the Physical Properties of *ABX*_3_ (*A* = K, Ag, Cu; *B* = Si*_x_*Ge*_y_*Sn*_z_*Pb_(1−*x*−*y*−*z*)_; *X* = Br, I) Metal Halide Perovskites

**DOI:** 10.3390/ma19061054

**Published:** 2026-03-10

**Authors:** Boyu Xie, Touwen Fan, Zixiong Ruan, Yue Hong, Xiongying He, Jianbo Chen

**Affiliations:** 1 School of Computer Science, Guangdong University of Technology, Guangzhou 510006, China; 2 Research Institute of Automobile Parts Technology, Hunan Institute of Technology, Hengyang 421002, China; 3 College of Physics and Electronic Engineering, Xingtai University, Xingtai 054000, China; 4 School of Science, Hunan Institute of Technology, Hengyang 421002, China

**Keywords:** metal halide perovskites, thermoelectric properties, medium-entropy local sublattices, first principles calculations, merit ZT value

## Abstract

The stability, elasticity, and thermoelectric property of *ABX*_3_ (*A* = K, Ag, Cu; *B* = Si*_x_*Ge*_y_*Sn*_z_*Pb_(1−*x*−*y*−*z*)_; *X* = Br, I) metal halide perovskites (MHPs) with *B*-type medium entropy sub-lattices (MESLs) are investigated by first principles calculations. The results show that the order of dissociation formation enthalpy ${\Delta H}_{f}$ for conventional unit cell *A*Pb*X*_3_ with changing atomic type in the *A* site is K < Ag < Cu, and for each case Br < I. The ${\Delta H}_{f}$ values of (K*B*Br_3_, K*B*I_3_, Ag*B*Br_3_) and (Cu*B*Br_3_, Cu*B*I_3_, Ag*B*I_3_) with MESL in the *B* site slightly increase and decrease, respectively, with the exception of certain situations. By using Slack’s model, the lattice thermal conductivity (LTC) $\kappa_{l}$ at finite temperatures is obtained. It is found that the LTC $\kappa_{l}$ for all MHPs shows an extremely low value at room temperature, not exceeding 1.5 Wm^−1^K^−1^. Interestingly, it is also found that the *B*-type MESLs significantly increase the ZT_max_ values of KPb*X*_3_, whereas they decrease the ZT_max_ values of CuPb*X*_3_ and AgPb*X*_3_, except for in some cases. All calculated parameters show obvious variation laws with the increase in atomic number of the high-content *B*-type atom in the *ABX*_3_, and Cu*BX*_3_ and Ag*BX*_3_ materials exhibit an extremely low ZT value (ZT ≈ 0) due to their high *σ* accompanied by high $\kappa_{e}$ and low *S*. We believe that KSi_0.375_Ge_0.25_Sn_0.25_Pb_0.125_Br_3_ with a ZT value of 3.012 can serve as an excellent thermoelectric material at room temperature. These findings make contributions to the design of high-quality thermoelectric MHP materials.

## 1. Introduction

In recent years, the issue of energy consumption has attracted a growing amount of attention. As a result, the development of high-performance thermoelectric generators that can capture heat from household heating, automobile exhaust, and industrial processes and directly convert it into electrical energy is an effective approach to improve energy utilization efficiency [1,2,3,4]. Thermoelectric generators have the ability to convert waste heat into electricity through the Seebeck effect. The power conversion efficiency of these generators is primarily determined by the properties of thermoelectric materials, and the dimensionless figure of merit (ZT) acts as the crucial metric for assessing thermoelectric properties [5,6,7]. The merit ZT can be calculated by the formula $ZT=\sigma S^{2}T/\kappa$, where *S*, *σ*, and κ represent the Seebeck coefficient, electrical conductivity, and total thermal conductivity, respectively.

Among numerous thermoelectric materials, the newly emerging metal halide perovskites (MHPs) have captured widespread interest as they exhibit superior carrier mobility and ultra-low thermal conductivity (TC) [8,9]. The typical general formula of the MHPs family is *ABX*_3_. Here, *A* stands for monovalent cations like Cs and Rb, *B* denotes divalent cations (Pb or Sn), and *X* refers to halogen anions (I, Br) [10,11]. Nevertheless, the application of CsPbI_3_ perovskite materials is subject to several limitations. Firstly, the perovskite phase exhibits poor stability at room temperature and can only be formed under high-temperature conditions. Secondly, the addition of Pb atoms is toxic [12,13,14]. Although the cubic crystal of CsPbBr_3_ demonstrates stability at ambient conditions, it remains unsuitable for manufacturing electronic devices because of its wide bandgap [15].

Over the past few years, researchers have employed several strategies involving atom doping and configuration exploration to enhance the stability, mechanical properties, and thermoelectric performance of MHP materials. Yamamoto et al. [16] first used the cluster expansion method (CEM) to explore partially substituted stable structures Cs(*B*, *B*′)I_3_ (*B*, *B*′ = Ge, Sn, and Pb), and further evaluated the transport properties of Cs(*B*, *B*′)I_3_ by first principles calculations. They pointed out that the steep density of states (DOS) gradient at the conduction band edge of all compositions endows N-type systems with a large Seebeck coefficient, enabling them to exhibit excellent thermoelectric performance, and when Cs(Ge, Sn)I_3_ solid solutions have a higher Ge content, their thermoelectric figure of merit ZT values are likely to be superior, reaching nearly 0.5 under room temperature conditions. Sharma et al. [17] reported the thermoelectric properties and stability of basic configurations of CsPbI_3_ and CsPbBr_3_ and the other two mixed substitution structures of CsPbBrI_2_ and CsPbIBr_2_ using first principles (FPs) calculations combining with Boltzmann transport. Their results showed that an increase in Br concentration can significantly decrease the formation energy of cubic MHPs from −463.52 KJ/mol to −604.84 KJ/mol. Nevertheless, there is little difference in the ZT values of these structures, with all being close to 0.75. Yan et al. [18] presented a combination of first-principles calculations and the Boltzmann transport theory (BTE) to discuss the carrier transport and thermoelectric performance of mixed MPH CsPb(I_1−*x*_Br*_x_*)_3_ with different Br compositions. The results demonstrated that the lattice thermal conductivity (LTC) of CsPb(I_1−*x*_Br*_x_*)_3_ is significantly suppressed compared to that of the pure counterparts due to strong mass disordering and strain fields upon halogen substitution, and the reconstruction of the conduction band of CsPb(I_1−*x*_Br*_x_*)_3_ leads to enhanced power factor, PF (PF = *S*^2^*σ*). These synergistic effects jointly contribute to the thermoelectric ZT of the MHP material reaching up to 1.7 at room temperature. Mumtaz et al. [12] inspected the structural, mechanical, electronic, optical, and thermoelectric response of all inorganic halide perovskite CsPb_1−*x*_Ge*_x_*Br_3_ (*x* = 0, 0.25, 0.50, 0.75, 1) MHPs to overcome the structural instability and toxic effects of high concentrations of Pb. It was observed that with the increase in Ge concentration, the elastic modulus of CsPb_1−*x*_Ge*_x_*Br_3_ increased, while its bandgap decreased. This indicates that doping is beneficial for enhancing mechanical properties and charge transport. Although a substantial body of prior research has endeavored to develop materials featuring outstanding properties such as high stability and favorable mechanical and thermoelectric characteristics, no ABX_3_ material has been engineered to achieve a satisfactory ZT value greater than 2 at room temperature, which falls significantly short of the requirements for practical material applications.

The design strategy of multi-component alloys has emerged as a core approach in the research and development of modern advanced materials, demonstrating significant potential to enhance material performance. Medium entropy materials (MEMs) are important members of the multi-component alloy family and achieve an optimal mixing entropy Δ*S*_mix_, 1.0R ≤ ΔSmix ≤ 1.5R to reduces the Gibbs free energy according to the Gibbs law ∆G=∆H−T∆S [19], and the severely distorted lattices presented in the MEMs give rise to a strong phonon scattering effect. This effect cuts down the LTC $\kappa_{l}$ while maintaining the electronic thermal conductivity (ETC) $\kappa_{e}$, thereby enhancing the overall thermoelectric performance [20,21,22,23]. Thus, suitable MEMs exhibit favorable structural stability and mechanical and thermoelectric properties.

However, to the best of our knowledge, no research on the “medium entropy sub-lattice (MESL)” design in *ABX*_3_ perovskite materials has been reported thus far. Moreover, the associated physical properties of these materials and the underlying atomic-level mechanism remain largely unknown. In the past few decades, with a significant improvement in computer computational capabilities, material simulation calculations, such as FPs calculations, have become increasingly popular in the exploration of novel materials and the investigation of microscopic mechanisms, and have been widely applied in various research fields, including metals, semiconductors, and two-dimensional materials [5,24,25,26,27,28,29]. In this context, the current research focuses on the design and investigation of the stability, mechanical properties, and thermoelectric performance of novel *ABX*_3_ perovskite materials integrated with *B*-type MESL configurations. This research is based on the special quasi-random structure (SQS) method in conjunction with FPs calculations. The stability is analyzed through formation energy calculations of *ABX*_3_ MHPs, and then the elastic properties are investigated using the energy-strain method. Finally, the thermoelectric performance is investigated by combining the electron BTE and Slack’s model [30,31].

## 2. Computational Details

All the calculations in this work were carried out using the Vienna Ab Initio Simulation Package (VASP) with the version of 5.4.4 [32] based on the theoretical framework of density functional theory (DFT) [33]. The core–electron interactions were described by the projector-augmented wave (PAW) pseudopotentials, and the Perdew–Burke–Ernzerhof (PBE) functional within the generalized gradient approximation (GGA) [34] was used to describe the exchange–correlation interaction between electrons. In the process of calculation, the PAW_GGA pseudo-potentials for K_pv, Cu, Ag, (Si-Pb), Br and I were used, and the cutoff energy was set as 350 eV after adequate test.

**Figure 1:**
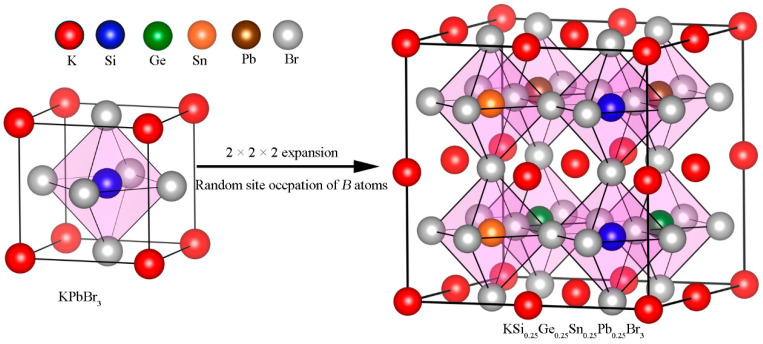
The model processing of KSi_0.25_Ge_0.25_Sn_0.25_Pb_0.25_Br_3_ structure with *B*-type MESL.

To establish the crystals of *ABX*_3_ (*A* = K, Ag, Cu; *B* = Si*_x_*Ge*_y_*Sn*_z_*Pb_(1−*x*−*y*−*z*)_; *X* = Br, I) MHP_S_ with *B-*type MESLs, a 2 × 2 × 2 supercell containing 40 atoms was initially created from the KPbBr_3_ unit cell with the cubic system (Pm-3m, space group No. 221). Subsequently, the *B-*type MESLs were constructed using the special quasi-random structure (SQS) method, keeping the type of atom in both *A* and *X* sites fixed, as carried out in the Alloy Theoretic Automated Toolkit (ATAT) with the version of 3.36 [35]. Figure 1 briefly depicts the model processing for realizing *B*-type MESLs in KSi_0.25_Ge_0.25_Sn_0.25_Pb_0.25_Br_3_. The K, Si, Ge, Sn, Pb and Br atoms are marked as red, blue, green, orange, brown and silvery balls, respectively, and Si, Ge, Sn and Pb are equimolar. During the structural optimization, 6 × 6 × 6 and 3 × 3 × 3 k-meshes with the Gamma-centered Monkhorst–Pack method were applied in *A*Pb*X*_3_ unit cells and *ABX*_3_ supercells, respectively.

The electronic transport properties were investigated by solving the Boltzmann equation (BTE), as implemented in the BoltzTraP2 codes [36], and denser 15 × 15 × 15 and 8 × 8 × 8 k-meshes were used for *A*Pb*X*_3_ and *ABX*_3_, respectively, to ensure the accuracy of calculations. We employed the constant relaxation time approximation (RTA) to calculate the ETC $\kappa_{e}$ derived from deformation potential (DP) theory [37,38], and the LTC $\kappa_{l}$ was calculated using Slack’s model [30,31].

## 3. Results and Discussion

### 3.1. Stability

The dissociation formation enthalpy ${\Delta H}_{f}$ can generally directly characterize the relative stability of multi-component materials, and it can be obtained from the energy difference between the total energy of multi-component compounds *E*_total_ and the sum of energy of stable elements, $\sum nE_{i}$, as follows [39,40,41,42]:

(1) $${\Delta H}_{f}=\frac{\left(E_{total}-\sum nE_{i}\right)}{N}$$

where *n* and *N* are the numbers of atoms of *i*th element and the total number of atoms for MHP materials, respectively. It should be noted that the ground-state selection in the current work is restricted to stable bulk phases. Nevertheless, the material synthesis process might entail the selection of specific compounds.

In this paper, 47 structures containing unit cells of *A*Pb*X*_3_ (*X* = Br, I) as well as supercells of *ABX*_3_ (*A* = K, Cu, Ag; *B* = Si*_x_*Ge*_y_*Sn*_z_*Pb_(1−*x*−*y*−*z*)_; *X* = Br, I) are selected as the focus of our investigation, and the detailed compositions are listed in the leftmost columns of Table S1. We firstly collect the lattice parameter *a*_0_ and dissociation formation enthalpy ${\Delta H}_{f}$ of CsPbBr_3_ and CsPbI_3_; the current results and previously reported data are summarized in Table 1. It can be seen that the values of *a*_0_ for CsPbBr_3_ and CsPbI_3_ are in good agreement with previously reported results [17,43,44]. Nevertheless, a maximum error of approximately 0.4 eV, ~20%, is observed for ${\Delta H}_{f}$ of CsPbBr_3_ and CsPbI_3_ when compared with the reported values [17]. This difference may come from the different configurations selected for the ground state.

**Figure 2:**
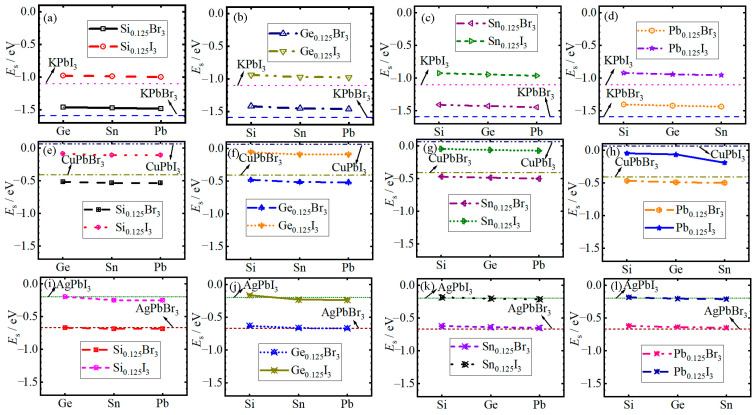
The dissociation formation enthalpy ${\Delta H}_{f}$ of (**a**–**d**) K*BX*_3_, (**e**–**h**) Cu*BX*_3_ and (**i**–**l**) Ag*BX*_3_ MHPs as a function of atomic number of the HCBA whose content exceeds 25 at.%. It should be noted that Si_0.125_Br_3_-Ge in 1, 2 and 3 rows represent the MHPs KSi_0.125_Ge_0.375_Sn_0.25_Pb_0.25_Br_3_, CuSi_0.125_Ge_0.375_Sn_0.25_Pb_0.25_Br_3_, and AgSi_0.125_Ge_0.375_Sn_0.25_Pb_0.25_Br_3_, respectively.

The calculated *a*_0_ and ${\Delta H}_{f}$ of *A*PbBr_3_, *A*PbI_3_, *AB*Br_3_ and *AB*I_3_ MHPs are also listed in Table S1. It can be clearly observed that the order of dissociation formation enthalpy ${\Delta H}_{f}$ for the conventional unit cell *A*Pb*X*_3_ with changing atomic type in the *A* site is K < Ag < Cu, and for each case Br < I, that is, KPbBr_3_ < KPbI_3_ < AgPbBr_3_ < AgPbI_3_ < CuPbBr_3_ < CuPbI_3_. Furthermore, it can be found that the *B*-type MESLs slightly increase and decrease the ${\Delta H}_{f}$ values of (K*B*Br_3_, K*B*I_3_, Ag*B*Br_3_) and (Cu*B*Br_3_, Cu*B*I_3_, Ag*B*I_3_), respectively, except for AgSi_0.125_Ge_0.25_Sn_0.375_Pb_0.25_Br_3_, AgSi_0.125_Ge_0.25_Sn_0.25_Pb_0.375_Br_3_, Ag Si_0.375_Ge_0.125_ Sn_0.25_Pb_0.25_I_3_, and AgSi_0.375_Ge_0.25_Sn_0.125_Pb_0.25_I_3_. To clearly show the variation trend in ${\Delta H}_{f}$, we plotted the Δ*H*_f_ of *ABX*_3_ as a function of the atomic number of high contents of the *B*-site atom (HCBA), whose content exceeds 25 at.%. As can be found in Figure 2a–l, the Δ*H*_f_ for all cases slightly decreases as the atomic number of HCBA increases.

### 3.2. The Lattice Thermal Conductivity

The total thermal conductivity of a material is generally divided into two core components that correspond to the two carriers of heat transfer: electrons and phonons. Therefore, the total thermal conductivity (TC) is calculated by $\kappa =\kappa_{l}+\kappa_{e}$. In this work, the Slack model was employed for calculating LTC $\kappa_{l}$, which is dependent on two key parameters, namely, the Debye temperature *θ*_D_ and the Grüneisen parameter *γ*, as follows [31,45]:

(2) $$\kappa_{l}\left(\theta_{\alpha }\right)=\frac{0.849\times 3\sqrt[{3}]{\left(4\right)}}{20\pi^{3}\left(1-0.154\gamma^{-1}+0.228\gamma^{-2}\right)}\times {\left(\frac{\kappa_{B}\theta_{\alpha }}{\hbar }\right)}^{2}\frac{\kappa_{B}M_{av}V^{\frac{1}{3}}}{\hbar \gamma^{2}}$$

and

(3) $$\kappa_{l}\left(T\right)=\kappa_{l}\left(\theta_{\alpha }\right)\frac{\theta_{\alpha }}{T}$$

where *V*, *M*_av_, $\theta_{\alpha }$ denote the primitive cell volume, average atomic mass, and acoustic-mode Debye temperature, respectively. Note: *θ*_D_ and *γ* are obtained by the Debye model carried out in the Gibbs2 codes [46].

We obtained the LTC $\kappa_{l}$ of all cases in accordance with Equations (2) and (3), and compiled the Grüneisen parameter *γ*, Debye temperature *θ*_D_ and LTC $\kappa_{l}$ at room temperature in Table 2 and Table S2. Clearly, it can be seen that the Debye temperature *θ*_D_ of 148.83 and 122.78 K for CsPbBr_3_ and CsPbI_3_ show good agreement with the theoretical values of 136.80 and 116.00 K, respectively, reported by Maleka et al. [47]. The calculated LTC $\kappa_{l}$ values of 0.54 for CsPbBr_3_ and 0.40 Wm^−1^K^−1^ for CsPbI_3_ are slightly larger and lower, respectively, than the experiment values of 0.42 ± 0.04 and 0.45 ± 0.05 Wm^−1^K^−1^. Both values are slightly higher than the corresponding results of 0.28 W m^−1^ K^−1^ for CsPbBr_3_ and 0.19 W m^−1^ K^−1^ for CsPbI_3_ obtained from the molecular dynamics (MD) calculations, and the value of 0.40 Wm^−1^K^−1^ for CsPb is also slightly higher than 0.25 Wm^−1^K^−1^ from the phonon Boltzmann equation (PBE) [48,49]. At room temperature, the type *B* MESLs would marginally increase the $\kappa_{l}$ of both *A*PbBr_3_ and *A*PbI_3_, except for CuSi_0.125_Ge_0.25_Sn_0.25_Pb_0.375_I_3_ and CuSi_0.25_Ge_0.25_Sn_0.375_-Pb_0.125_I_3_. Moreover, the $\kappa_{l}$ of all MHPs exhibited an extremely low value at room temperature, not exceeding 1.5 Wm^−1^K^−1^.

**Figure 3:**
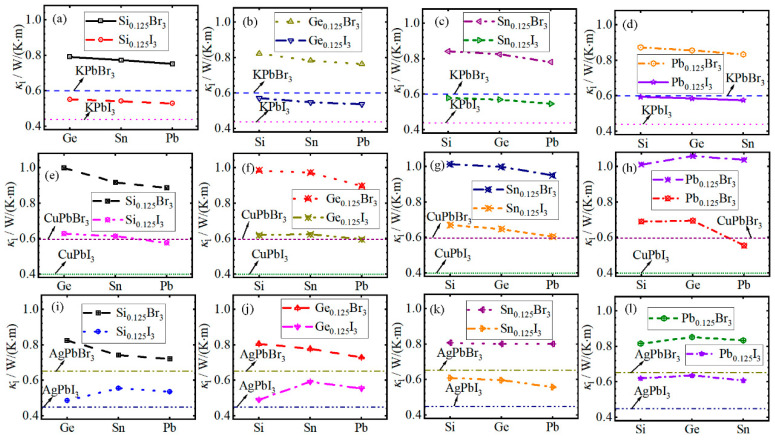
The lattice thermal conductivity $\kappa_{l}$ of (**a**–**d**) K*BX*_3_, (**e**–**h**) Cu*BX*_3_ and (**i**–**l**) Ag*BX*_3_ MHPs as a function of atomic number of high contents of *B* atoms.

Figure S1a–d presents the calculated $\kappa_{l}$ of all cases as the temperature increases to 1000 K. It is evident that the $\kappa_{l}$ decreases sharply in the temperature range of 100 to 300 K, after which the rate of decrease slows down. Moreover, the $\kappa_{l}$ of *A*PbBr_3_ and *AB*Br_3_ is larger than that of *A*PbI_3_ and *AB*I_3_ across the entire temperature range. Additionally, the $\kappa_{l}$ of *AB*Br_3_ and *AB*I_3_ as a function of the atomic number of HCBA is plotted in Figure 3a–l. The results indicate that the $\kappa_{l}$ of *AB*Br_3_ and *AB*I_3_ slightly decreases with the increase in the atomic number of HCAB, with the exception of CuSi*_x_*Ge*_y_*Sn*_z_*Pb_0.125_*X*_3_, AgSi_0.125_Ge*_y_*Sn*_z_*Pb_(0.875−*y*−*z*)_I_3_, AgSi*_x_*Ge_0.125_Sn*_z_*Pb_(0.875-*x*-*z*)_I_3_ and AgSi*_x_*Ge*_y_*Sn*_z_*Pb_0.125_*X*_3_. The $\kappa_{l}$ values of CuSi*_x_*Ge*_y_*Sn*_z_*Pb_0.125_*X*_3_ and AgSi*_x_*Ge_y_Sn*_z_*Pb_0.125_*X*_3_ first increase from the HCBA of Si to Ge, and then decrease to Sn. In contrast, the $\kappa_{l}$ values of AgSi_0.125_Ge*_y_*Sn*_z_*Pb_(0.875−*y*−*z*)_I_3_ and AgSi*_x_*Ge_0.125_Sn*_z_*Pb_(0.875−*x*−*z*)_I_3_, respectively, increase from the HCBA of Ge to Sn and Si to Sn, and then decrease to Pb.

### 3.3. Electrical Transport Properties

At present, electronic transport calculations are mainly performed by solving the electron Boltzmann transport theory (EBTP). The Seebeck coefficients *S* and *σ*, as important indicator parameters for evaluating the thermoelectric properties of *ABX*_3_, can be calculated by integrating the energy-projected transport distribution tensor *σ_αβ_*, and the p- and n-types MHPs are determined by a negative and positive chemical potential *μ*, respectively, as follows:

(4) $$\sigma_{\alpha \beta }(T,\mu )=\frac{1}{\Omega }\int \sigma_{\alpha \beta }(\varepsilon )\left[-\frac{\partial {ƒ}_{0}(T,\varepsilon ,\mu )}{\partial \varepsilon }\right]d\varepsilon$$

(5) $$S_{\alpha \beta }(T,\mu )=\frac{1}{eT\Omega \sigma_{\alpha \beta }(T,\mu )}\int \sigma_{\alpha \beta }(\varepsilon )(\varepsilon -\mu )\left[-\frac{\partial {ƒ}_{0}(T,\varepsilon ,\mu )}{\partial \varepsilon }\right]d\varepsilon$$

where *e* and *ε* are the electronic charge and band-energy, respectively.

The *σ_αβ_* including the system-dependent information be expressed as follows:

(6) $$\sigma_{\alpha \beta }(\varepsilon )=\frac{e^{2}}{N}\sum_{i,K}\tau_{i,k}\nu_{\alpha }(i,K)\nu_{\beta }(i,K)\frac{\delta (\varepsilon -\varepsilon_{i,K})}{d\varepsilon }$$

where *N*, *i*, *K* and *ν_α_*_, *β*_ (*i*, *K*) are the number of k-points, the band index, the wave vector and the group velocity of the acoustic wave, respectively.

In Equation (5), the electronic relaxation time *τ* is solved by the deformation potential (DP) theory combined with the rigid band approximation (RBA) method for semiconductor materials [51,52]. Using the DP theory, *τ* can be calculated by:

(7) $$\tau =\frac{2\sqrt{2\pi }\hbar^{4}C}{3{\left(\kappa_{B}Tm^{*}\right)}^{\frac{3}{2}}E_{dp}^{2}}$$

where $C=\left[\frac{\partial^{2}E}{\partial {\left(\frac{∆a}{a_{0}}\right)}^{2}}\right]/V$, $m^{*}=\hbar^{2}/\left[\partial^{2}\varepsilon /\partial K^{2}\right]$ and $E_{dp}=dE_{edg}/d\left(\frac{∆a}{a_{0}}\right)$ are the elastic constant, effective mass and DP constant; *E*, *a*_0_, *V*, and *E*_edg_ are the total energy (including strain energy), lattice constant, volume and band edge energy, respectively.

Based on the Wiedemann–Franz (WF) law, the key thermoelectric parameters, that is, the $\kappa_{e}$ of *A*Pb*X*_3_ MHPs, can be obtained through the determined *σ* [36]:

(8) $$\kappa_{e}=L\sigma T$$

where $L=\frac{\pi^{2}\kappa_{B}^{2}}{3e^{2}}$ is the Lorentz constant.

According to Equation (7), we first calculated the energy band structure of *A*Pb*X*_3_ unit cells and *A*Si_0.25_Ge_0.25_Sn_0.25_Pb_0.25_*X*_3_ supercells along the path G-X-M-G-R-X of the highly symmetric points in the Brillouin zone, where G, X, M, and R are (0, 0, 0), (0, 0.5, 0), (0.5, 0.5, 0), and (0.5, 0.5, 0.5), respectively; the results are plotted in Figures S2 and S3. In Figure S2, it can be observed that the energy band structures of *A*Pb*X*_3_ (A = Cs, K) clearly indicate semiconductor characteristics due to the presence of a distinct band gap. Moreover, the band gaps of 1.76 eV for CsPbBr3 and 1.47 eV for CsPbI3 are in good agreement with those reported in other studies [12,17]. At the G point, the values of the conduction band for *A*Pb*X*_3_ (A = Cu, Ag) show obvious negative numbers of −0.27 and −0.23 eV, respectively, and there is either band overlap or the conduction band and valence band are very close to each other, indicating that they demonstrate semi-metal characteristics [53]. In Figure S3, the band gaps of 0.33 and 0.20 eV for KSi_0.25_Ge_0.25_Sn_0.25_Pb_0.25_Br_3_ and KSi_0.25_Ge_0.25_Sn_0.25_Pb_0.25_I_3_ are lower than the 1.72 and 1.44 eV for KPbBr_3_ and KPbI_3_. Meanwhile, *A*Si_0.25_Ge_0.25_Sn_0.25_Pb_0.25_*X*_3_ (A = Cu, Ag) exhibit more band overlaps, showing obvious metallic characteristics. This indicates that the *B*-type MESLs would excite more carriers to participate in conduction, directly enhancing the ETC *σ*. But Equation (7) cannot be used to calculate the electronic relaxation time *τ* for *ABX*_3_ (A = Cu, Ag) due to the materials exhibiting obvious metallic characteristics. Thus, Equation (6) is solved under constant relaxation time approximation (RTA) [54,55], and for simplicity, in this study, we adopted a relaxation time of 10^−13^ s for *ABX*_3_ (A = Cu, Ag).

The detailed calculation results, including *C*, *E*_dp_ (eV), and *τ*, are listed in Table S3, and the τ of 0.048 ps for CsPbI_3_ has a good consistency with the experimental value of 0.022 ps. We further obtained the ZT values of all *ABX*_3_ MHPs within the *μ* range of −0.28 to 0.28 eV, approaching to Fermi energy, and collected the *S*, *σ*, power factor PF (PF = *S*^2^*σ*) and maximum ZT_max_ values in Table 3 and Table S4 at room temperature. It can be seen that the *S* and ZT_max_ values of 244.34 and 261.21 μV/K and the 0.88 and 1.05 found for CsPbBr_3_ and CsPbI_3_ are in good agreement with previous theoretical values of ~280 and ~260 μV/Kand ~0.74 and ~0.65 at room temperature [17,49,56]. Furthermore, the type-*B* MESLs significantly increased the ZT_max_ values, reaching approximately 2.383 for KPbBr_3_, while slightly decreasing the ZT_max_ values of CuPb*X*_3_ and AgPb*X*_3_, with the exception of a few scattered data points. The results further show that Cu*BX*_3_ and Ag*BX*_3_ materials exhibit an extremely low ZT value (ZT ≈ 0) because of their high *σ* accompanied by high $\kappa_{e}$ and low *S*, which is a common characteristic of metallic materials [57]. Hence, these materials are not suitable candidates for thermoelectric materials.

In accordance with Equation (7), we supplemented the variation in *τ*_p_ of *A*Pb*X*_3_ (A = Cs, K) and KSi_0.25_Ge_0.25_Sn_0.25_Pb_0.25_*X*_3_ MHPs in the range of 100–1000 K in Figure S4a, and calculated the corresponding change in ZT values with temperature increasing, which is plotted in Figure S4b. It can be seen that the *τ*_p_ of all cases decreased rapidly as the temperature increased to 300 K, followed by a gradual reduction. The *τ*_p_ of approximately 5.18 ps for KSi_0.25_Ge_0.25_Sn_0.25_Pb_0.25_*X*_3_ is larger than that for *A*Pb*X*_3_ within the investigated temperature range. The ZT values of *A*Pb*X*_3_ (A = Cs, K) increase linearly to approximately 2.809 as the temperature rises. In contrast, the ZT values of KSi_0.25_Ge_0.25_Sn_0.25_Pb_0.25_*X*_3_ initially increase linearly to a maximum value of about 1.773 with the increase in temperature. Subsequently, the ZT values show a softening trend after 300 K and 600 K. It has been verified that there is an optimal temperature region for the ZT value [58].

**Figure 4:**
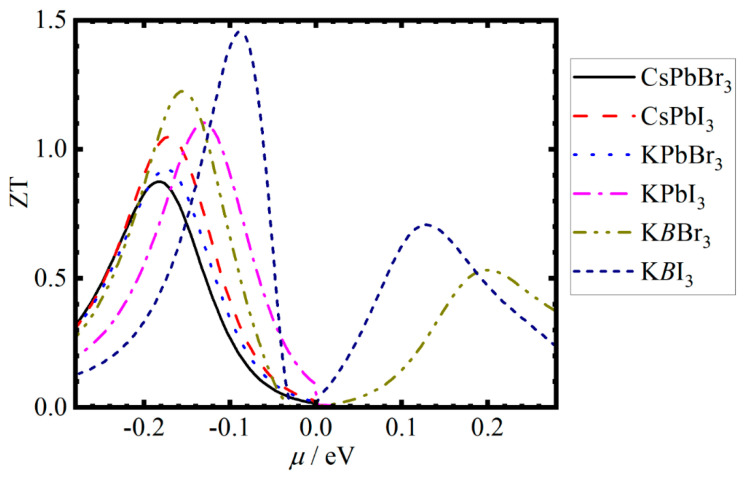
The ZT of *A*PbX_3_ and KSi_0.25_Ge_0.25_Sn_0.25_Pb_0.25_*X*_3_ MHPs as a function of chemical potential.

**Figure 5:**
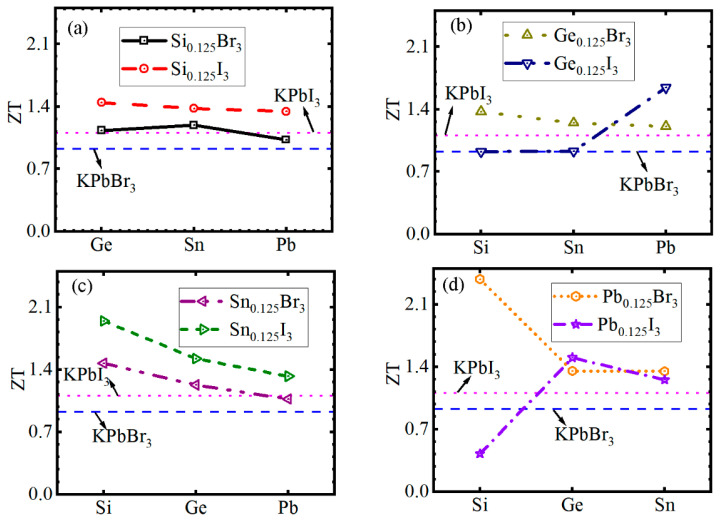
The ZT of (**a**–**d**) K*BX*_3_ MHPs as a function of the atomic number of HCBA.

We additionally plotted the ZT curves of *A*Pb*X*_3_ and *A*Si_0.25_Ge_0.25_Sn_0.25_Pb_0.25_*X*_3_ MHPs (*A* = Cs and K) as the *μ* value increases in Figure 4. Evidently, the ZT curves exhibit unimodal or multimodal patterns near the Fermi level. To investigate a potential relationship between the ZT_max_ value and the atomic fraction configurations, Figure 5a–d show the ZT_max_ of K*BX*_3_ with the increase in the atomic number of HCBA. It is found that with the increase in the atomic number of HCAB, the ZT_max_ values of KSi_0.125_Ge*_y_*Sn*_z_*Pb_(0.875−*y*−*z*)_I_3_, KSi*_x_*Ge_0.125_Sn*_z_*Pb_(0.875−_*_x_*_−*z*)_Br_3_ and KSi*_x_*Ge*_y_*Sn_0.125_Pb_(0.875−_*_x_*_−_*_y_*_)_X_3_ decrease, whereas the ZT values of the rest of the cases present fluctuating changes. Among all MHPs, the KSi_0.375_Ge_0.25_Sn_0.25_Pb_0.125_Br_3_ showed a larger ZT value of 2.383, demonstrating that it can serve as an excellent thermoelectric material at room temperature.

The PBE functional typically underestimates the band gap. In contrast, the Heyd–Scuseria–Ernzerhof 2006 (HSE06) hybrid functional is commonly utilized to precisely calculate the band structure, yielding more reliable outcomes for electronic transport properties. We utilized the HSE06 hybrid functional to compute the critical KPbBr_3_ and KSi_0.375_Ge_0.25_Sn_0.25_Pb_0.125_Br_3_, and the key values of *S*, *σ*, PF and ZT_max_ are summarized in Table S4. The results indicate that the ZT values of 0.845 and 3.012 for KPbBr_3_ obtained from HSE06 are slightly lower and higher, respectively, than the calculated values of 0.922 and 2.383 from PBE.

## 4. Conclusions

In this work, the stability and thermoelectric properties of *ABX*_3_ (*A* = K, Ag, Cu; *B* = Si, Ge, Sn, Pb; *X* = Br, I) MHPs are investigated by first principles calculations. Based on a comprehensive analysis of stability and thermoelectric properties, the target parameters of all calculations exbibit obvious variation laws with the increases in atomic number, and Cu*BX*_3_ and Ag*BX*_3_ materials exhibit an extremely low ZT value (ZT ≈ 0) due to their high *σ* accompanied by high $\kappa_{e}$ and low *S*. We believe that the ZT values of 3.012 for KSi_0.375_Ge_0.25_Sn_0.25_Pb_0.125_Br_3_ can serve as excellent thermoelectric materials at room temperature. The main results are as follows:

1. The results show that the order of dissociation formation enthalpy ${\Delta H}_{f}$ for the conventional unit cell *A*Pb*X*_3_ with a changing atomic type in the *A* site is K < Ag < Cu, and for each case, Br < I. The ${\Delta H}_{f}$ values of (K*B*Br_3_, K*B*I_3_, Ag*B*Br_3_) and (Cu*B*Br_3_, Cu*B*I_3_, Ag*B*I_3_) with MESL in the *B* site slightly increase and decrease, respectively, with the exception of certain situations.
2. By increasing the atomic number of the *B*-type atom, the content of which exceeds 25 at.%, namely HCBA, Δ*H*_f_ slightly decreases.
3. At room temperature, the type-*B* MESLs would slightly increase the $\kappa_{l}$ of *A*PbBr_3_ and *A*PbI_3_, except for CuSi_0.125_Ge_0.25_Sn_0.25_Pb_0.375_I_3_ and CuSi_0.25_Ge_0.25_Sn_0.375_Pb_0.125_I_3_, and the $\kappa_{l}$ for all MHPs shows an extremely low $\kappa_{l}$ at room temperature, no more than 1.5 Wm^−1^K^−1^.
4. The $\kappa_{l}$ of *AB*Br_3_ and *AB*I_3_ slightly decreases when the atomic number of the *B*-type atom increased, except for a few scattered points.
5. The type-*B* MESLs significantly increase the ZT_max_ values of KPb*X*_3_ while decreasing the ZT_max_ values of CuPb*X*_3_ and AgPb*X*_3_, except for several values.
6. With the increase in atomic number of the type-*B* atom, the ZT_max_ values of KSi_0.125_Ge*_y_*Sn*_z_*Pb_(0.875−*y*−*z*)_I_3_, KSi*_x_*Ge_0.125_Sn*_z_*Pb_(0.875−_*_x_*_−*z*)_Br_3_ and KSi*_x_*Ge*_y_*Sn_0.125_Pb_(0.875−_*_x_*_−_*_y_*_)_X_3_ decrease, whereas the ZT values of the rest of the cases present fluctuating changes. Among all MHPs, the KSi_0.375_Ge_0.25_Sn_0.25_Pb_0.125_Br_3_ shows larger ZT values of 3.012, meaning that it can serve as an excellent thermoelectric material at room temperature.

## Figures and Tables

**Table 1:** The calculated lattice constants *a*_0_ and the dissociation enthalpy ${\Delta H}_{f}$.

Compounds	*a*_0_ (Å)	∆*H*_f_ (eV/atom)
CsPbBr_3_	5.989; 5.874 [17]; 5.989 [43]; 5.966 [44]	−1.695; −1.254 [17]
CsPbI_3_	6.385; 6.242 [17]; 6.383 [43]; 6.376 [44]	−1.211; −0.961 [17]

**Table 2:** The calculated Grüneisen parameter *γ*, Debye temperature *θ*_D_ (K) and lattice thermal conductivity $\kappa_{l}$ (Wm^−1^K^−1^).

Compounds	*γ*	*θ*_D_	$\kappa_{l}$
CsPbBr_3_	2.240	148.83 136.80 [47]	0.54 0.42 ± 0.04 [50]; 0.28 [48]
CsPbI_3_	2.238	122.78 116.00 [47]	0.40 0.45 ± 0.05 [50]; 0.25 [49]; 0.19 [48]

**Table 3:** The Seebeck coefficient, *S* (μV/K); electric conductivity, *σ* (10^4^ Ω^−1^m^−1^); power factor, PF (10^−3^ WK^−2^m^−1^); and figure of merit, ZT.

	*S*	*σ*	PF	ZT
CsPbBr_3_	244.34 242 [17]; 280.00 [56]	4.09	2.44	0.88 0.74 [17]
CsPbI_3_	261.21 199 [17]; 260.00 [56]	3.29	2.25	1.05 0.66 [17]; 0.65 [49]

## Data Availability

The original contributions presented in this study are included in the article/Supplementary Material. Further inquiries can be directed to the corresponding authors.

## Supplementary Materials

The following supporting information can be downloaded at: https://www.mdpi.com/article/10.3390/ma19061054/s1, Table S1: The calculated lattice constants *a*_0_ (Å) and the dissociation formation energy ${\Delta H}_{f}$ (eV/atom); Table S2: The calculated Grüneisen parameter *γ*, Debye temperature *θ*_D_ (K) and lattice thermal conductivity (LTC) $\kappa_{l}$ (Wm^−1^K^−1^); Figure S1: The calculated $\kappa_{l}$ of (a) *A*Pb*X*_3_, (b) K*BX*_3_, (c) Cu*BX*_3_ and (d) Ag*BX*_3_ as a function of temperature; Figure S2: (a–h) The energy band curves of *A*Pb*X*_3_ unit cells; Figure S3: (a–f) The energy band curves of *AB*^eq^*X*_3_ unit cells; Table S3: The elastic module *C* (GPa), DP constant *E*_dp_ (eV), effective mass *m*∗ (m_0_), and relaxation time *τ* (ps) of under n-type and p-type doping at 300 K; Table S4: The Seebeck coefficient *S* (μV/K), electric conductivity *σ* (10^5^ Ω^−1^m^−1^), power factor PF (10^−3^WK^−2^m^−1^) and figure of merit ZT_max_. Figure S4: (a) The relaxation time *τ*_p_ and (b) ZT values of *A*Pb*X*_3_ (A = Cs, K) and KSi_0.25_Ge_0.25_Sn_0.25_Pb_0.25_*X*_3_ MHPs as a function of temperature, respectively.
