# Supplementary material for: First-Principles Investigation of the Effects of B-Type Medium Entropy Local Sublattice on the Physical Properties of ABX3 (A = K, Ag, Cu; B = SixGeySnzPb(1−x−y−z); X = Br, I) Metal Halide Perovskites
Source: Materials (Basel). 2026 Mar 10;19(6):1054. doi: 10.3390/ma19061054 (PMC13027416; doi:10.3390/ma19061054)
Supplement: Supplementary file 1 [file materials-19-01054-s001.zip › materials-4130522-supplementary.pdf]

Table S1. The calculated lattice constants a_0 (Å) and the dissociation formation energy ΔH_f (eV/atom). Note: The value of * is derived from a 3×2×2 supercell containing 60 atoms.

Compounds	K		Cu		Ag	
	a_0	E_f	a_0	E_f	a_0	E_f
APbBr$_3$	5.954	−1.588	5.854	−0.404	5.856	−0.668
APbI$_3$	6.357	−1.100	6.227	0.064	6.263	−0.194
AB^{eq}Br$_3$	5.704	−1.439; −1.457*	5.580	−0.497	5.647	−0.648
AB^{eq}I$_3$	6.102	−0.958	5.948	−0.073	6.010	−0.213
ASi$_{0.125}$Ge$_{0.375}$(SnPb)$_{0.5}$Br$_3$	5.712	−1.460	5.629	−0.515	5.672	−0.667
ASi$_{0.125}$Sn$_{0.375}$(GePb)$_{0.5}$Br$_3$	5.763	−1.473	5.650	−0.532	5.679	−0.684
ASi$_{0.125}$Pb$_{0.375}$(GeSn)$_{0.5}$Br$_3$	5.774	−1.480	5.656	−0.531	5.685	−0.685
ASi$_{0.125}$Ge$_{0.375}$(SnPb)$_{0.5}$I$_3$	6.112	−0.978	5.948	−0.086	6.038	−0.196
ASi$_{0.125}$Sn$_{0.375}$(GePb)$_{0.5}$I$_3$	6.160	−0.991	6.051	−0.109	6.065	−0.249
ASi$_{0.125}$Pb$_{0.375}$(GeSn)$_{0.5}$I$_3$	6.172	−0.997	6.060	−0.109	6.072	−0.250
AGe$_{0.125}$Si$_{0.375}$(SnPb)$_{0.5}$Br$_3$	5.692	−1.418	5.576	−0.479	5.628	−0.629
AGe$_{0.125}$Sn$_{0.375}$(SiPb)$_{0.5}$Br$_3$	5.745	−1.451	5.625	−0.513	5.653	−0.664
AGe$_{0.125}$Pb$_{0.375}$(SiSn)$_{0.5}$Br$_3$	5.754	−1.458	5.678	−0.515	5.683	−0.667
AGe$_{0.125}$Si$_{0.375}$(SnPb)$_{0.5}$I$_3$	6.089	−0.937	5.931	−0.054	6.007	−0.162
AGe$_{0.125}$Sn$_{0.375}$(SiPb)$_{0.5}$I$_3$	6.142	−0.970	6.014	−0.091	6.039	−0.230
AGe$_{0.125}$Pb$_{0.375}$(SiSn)$_{0.5}$I$_3$	6.153	−0.976	6.115	−0.092	6.130	−0.232
ASn$_{0.125}$Si$_{0.375}$(GePb)$_{0.5}$Br$_3$	5.643	−1.405	5.579	−0.469	5.577	−0.620
ASn$_{0.125}$Ge$_{0.375}$(SiPb)$_{0.5}$Br$_3$	5.683	−1.426	5.578	−0.485	5.632	−0.637
ASn$_{0.125}$Pb$_{0.375}$(SiGe)$_{0.5}$Br$_3$	5.709	−1.445	5.652	−0.499	5.673	−0.650
ASn$_{0.125}$Si$_{0.375}$(GePb)$_{0.5}$I$_3$	6.043	−0.923	6.024	−0.044	6.016	−0.184
ASn$_{0.125}$Ge$_{0.375}$(SiPb)$_{0.5}$I$_3$	6.079	−0.944	5.947	−0.060	5.992	−0.201
ASn$_{0.125}$Pb$_{0.375}$(SiGe)$_{0.5}$I$_3$	6.112	−0.963	6.025	−0.072	6.026	−0.213
APb$_{0.125}$Si$_{0.375}$(GeSn)$_{0.5}$Br$_3$	5.654	−1.401	5.517	−0.467	5.573	−0.617
APb$_{0.125}$Ge$_{0.375}$(SiSn)$_{0.5}$Br$_3$	5.659	−1.421	5.532	−0.488	5.582	−0.637
APb$_{0.125}$Sn$_{0.375}$(SiGe)$_{0.5}$Br$_3$	5.682	−1.434	5.543	−0.496	5.603	−0.645
APb$_{0.125}$Si$_{0.375}$(GeSn)$_{0.5}$I$_3$	6.048	−0.920	5.889	−0.044	5.959	−0.183
APb$_{0.125}$Ge$_{0.375}$(SiSn)$_{0.5}$I$_3$	6.055	−0.939	5.906	−0.063	5.964	−0.202
APb$_{0.125}$Sn$_{0.375}$(SiGe)$_{0.5}$I$_3$	6.080	−0.953	6.145	−0.188	5.987	−0.210

Table S2. The calculated Grüneisen parameter γ, Debye temperature θ_D (K) and lattice thermal conductivity (LTC) κ_l ($Wm^{-1}K^{-1}$).

Compounds	K			Cu			Ag		
	γ	θ_D	κ_l	γ	θ_D	κ_l	γ	θ_D	κ_l
APbBr$_3$	2.208	162.43	0.60	2.095	154.30	0.60	2.019	150.46	0.65
APbI$_3$	2.211	131.47	0.44	2.189	125.93	0.40	2.094	123.82	0.45
AB^{eq}Br$_3$	2.058	186.43	0.81	2.056	196.67	0.99	2.167	185.60	0.83
AB^{eq}I$_3$	2.095	147.54	0.56	2.191	157.26	0.63	2.204	150.41	0.60
ASi$_{0.125}$Ge$_{0.375}$(SnPb)$_{0.5}$Br$_3$	2.071	185.12	0.79	2.026	193.67	1.00	2.158	183.60	0.82
ASi$_{0.125}$Sn$_{0.375}$(GePb)$_{0.5}$Br$_3$	2.072	182.47	0.77	2.107	192.87	0.92	2.242	181.66	0.74
ASi$_{0.125}$Pb$_{0.375}$(GeSn)$_{0.5}$Br$_3$	2.089	179.97	0.75	2.119	189.56	0.89	2.251	178.76	0.72
ASi$_{0.125}$Ge$_{0.375}$(SnPb)$_{0.5}$I$_3$	2.106	146.65	0.55	2.199	156.61	0.63	2.314	145.01	0.49
ASi$_{0.125}$Sn$_{0.375}$(GePb)$_{0.5}$I$_3$	2.102	144.77	0.54	2.177	153.23	0.61	2.240	147.47	0.56
ASi$_{0.125}$Pb$_{0.375}$(GeSn)$_{0.5}$I$_3$	2.117	143.20	0.53	2.206	150.30	0.58	2.260	145.46	0.53
AGe$_{0.125}$Si$_{0.375}$(SnPb)$_{0.5}$Br$_3$	2.045	187.84	0.82	2.049	196.62	0.98	2.173	185.17	0.81

$AGe_{0.125}Sn_{0.375}(SiPb)_{0.5}Br_3$	2.064	183.75	0.78	2.059	194.30	0.97	2.203	183.05	0.78
$AGe_{0.125}Pb_{0.375}(SiSn)_{0.5}Br_3$	2.076	181.18	0.76	2.109	190.62	0.90	2.241	179.80	0.73
$AGe_{0.125}Si_{0.375}(SnPb)_{0.5}I_3$	2.081	148.46	0.57	2.202	157.36	0.62	2.312	146.51	0.49
$AGe_{0.125}Sn_{0.375}(SiPb)_{0.5}I_3$	2.096	145.60	0.55	2.169	154.36	0.62	2.191	148.78	0.59
$AGe_{0.125}Pb_{0.375}(SiSn)_{0.5}I_3$	2.105	144.01	0.54	2.174	151.14	0.60	2.225	146.18	0.56
$ASn_{0.125}Si_{0.375}(GePb)_{0.5}Br_3$	2.040	190.45	0.84	2.061	201.01	1.01	2.192	187.74	0.81
$ASn_{0.125}Ge_{0.375}(SiPb)_{0.5}Br_3$	2.053	188.98	0.83	2.068	199.34	1.00	2.198	186.56	0.80
$ASn_{0.125}Pb_{0.375}(SiGe)_{0.5}Br_3$	2.077	183.84	0.78	2.054	191.31	0.95	2.176	181.41	0.80
$ASn_{0.125}Si_{0.375}(GePb)_{0.5}I_3$	2.087	150.24	0.58	2.158	160.00	0.67	2.184	152.01	0.61
$ASn_{0.125}Ge_{0.375}(SiPb)_{0.5}I_3$	2.098	149.26	0.57	2.182	158.75	0.65	2.200	151.07	0.60
$ASn_{0.125}Pb_{0.375}(SiGe)_{0.5}I_3$	2.112	145.90	0.55	2.178	152.66	0.60	2.229	147.36	0.56
$APb_{0.125}Si_{0.375}(GeSn)_{0.5}Br_3$	2.017	193.36	0.87	2.093	205.59	1.01	2.204	191.20	0.82
$APb_{0.125}Ge_{0.375}(SiSn)_{0.5}Br_3$	2.030	191.78	0.86	2.044	203.89	1.06	2.171	190.77	0.85
$APb_{0.125}Sn_{0.375}(SiGe)_{0.5}Br_3$	2.037	189.20	0.83	2.031	200.01	1.04	2.167	187.99	0.83
$APb_{0.125}Si_{0.375}(GeSn)_{0.5}I_3$	2.073	152.01	0.59	2.159	163.03	0.69	2.185	154.21	0.62
$APb_{0.125}Ge_{0.375}(SiSn)_{0.5}I_3$	2.082	151.00	0.58	2.151	162.14	0.69	2.171	154.13	0.64
$APb_{0.125}Sn_{0.375}(SiGe)_{0.5}I_3$	2.081	149.22	0.57	2.397	160.04	0.55	2.189	152.04	0.61

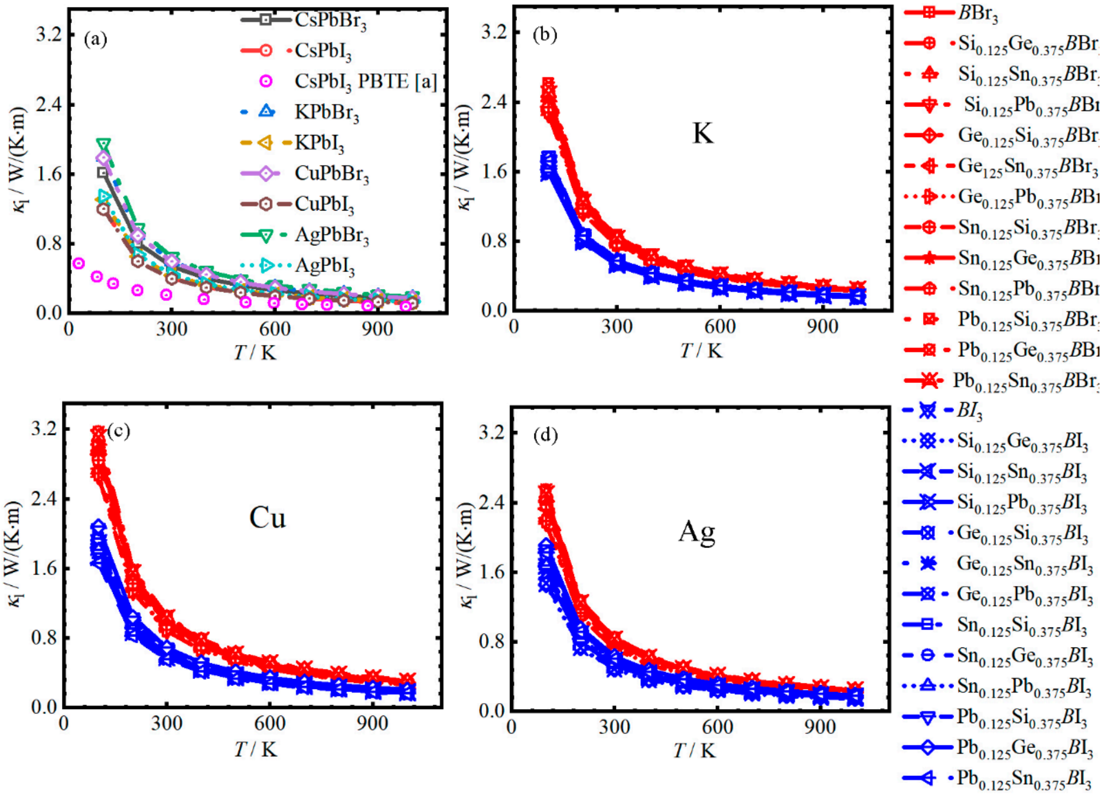

Figure S1. The calculated κ_l of (**a**) $APbX_3$, (**b**) KBX_3, (**c**) $CuBX_3$ and (**d**) $AgBX_3$ as a function of temperature.

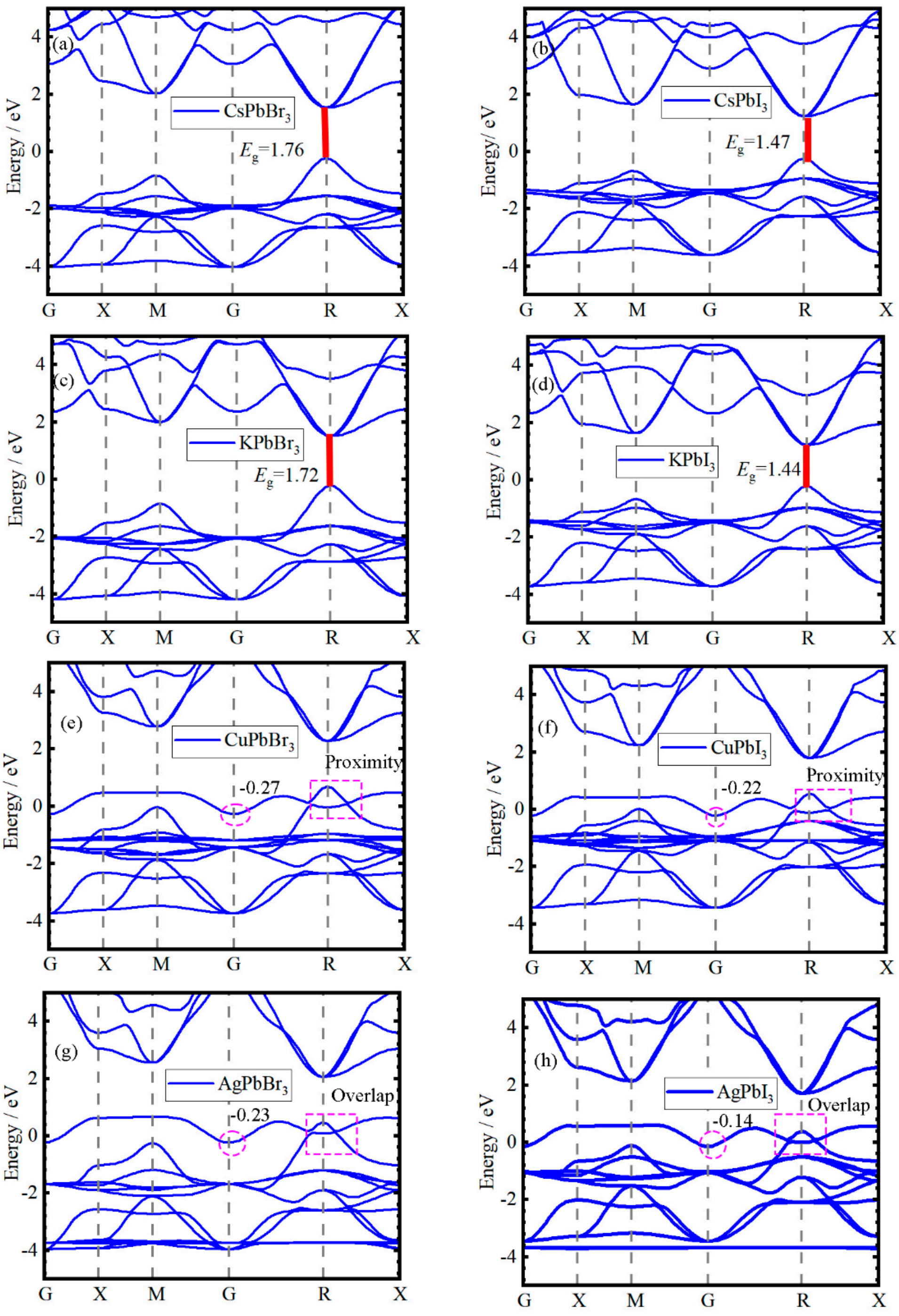

Figure S2. (**a–h**) The energy band curves of *A*Pb*X*3 unit cells.

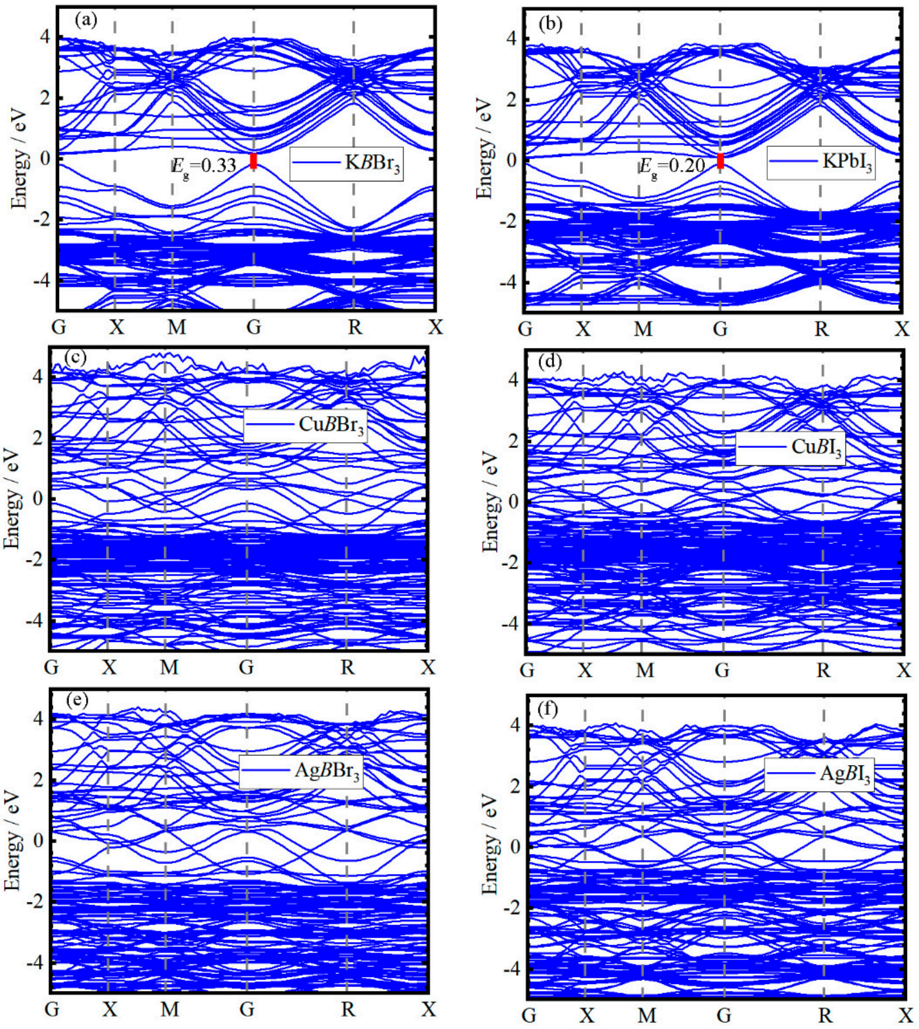

Figure S3. (**a–f**) The energy band curves of $AB^{eq}X_3$ unit cells.

Table S3. The elastic module C (GPa), DP constant E_{dp} (eV), effective mass $m*$ (m_0), and relaxation time τ (ps) of under n-type and p-type doping at 300 K.

	C	E_{dp} (hole)	E_{dp} (electron)	$m*$ (hole)	$m*$ (electron)	τ (hole)	τ (electron)
$CsPbBr_3$	44.64	10.67	7.93	0.175	0.832	0.187	0.033
$CsPbI_3$	35.90	10.43	8.25	0.152	0.525	0.194	0.048<br>0.022 (exp.)[1]
$KPbBr_3$	45.80	10.13	7.28	0.165	0.856	0.232	0.038
$KPbI_3$	36.67	9.94	7.66	0.145	0.767	0.235	0.032
$KB^{eq}Br_3$	48.18	12.19	7.61	0.057	1.281	0.831	0.020
$KB^{eq}I_3$	39.61	11.86	7.95	0.046	1.099	0.996	0.019
$CsSi_{0.125}Ge_{0.375}(SnPb)_{0.5}Br_3$	48.05	12.00	8.07	0.068	1.220	0.656	0.019
$CsSi_{0.125}Sn_{0.375}(GePb)_{0.5}Br_3$	47.22	11.83	6.60	0.065	1.444	0.711	0.022
$CsSi_{0.125}Pb_{0.375}(GeSn)_{0.5}Br_3$	46.63	11.89	6.03	0.081	1.544	0.499	0.023
$CsGe_{0.125}Si_{0.375}(SnPb)_{0.5}Br_3$	48.03	12.13	8.63	0.046	1.312	1.154	0.015
$CsGe_{0.125}Sn_{0.375}(SiPb)_{0.5}Br_3$	46.52	12.24	6.41	0.055	1.497	0.840	0.022

$CsGe_{0.125}Pb_{0.375}(SiSn)_{0.5}Br_3$	45.70	10.72	6.08	0.073	1.569	0.704	0.022
$CsSn_{0.125}Si_{0.375}(GePb)_{0.5}Br_3$	47.24	11.31	5.81	0.046	1.209	1.305	0.037
$CsSn_{0.125}Ge_{0.375}(SiPb)_{0.5}Br_3$	48.29	11.93	7.72	0.058	1.220	0.847	0.021
$CsSn_{0.125}Pb_{0.375}(SiGe)_{0.5}Br_3$	46.49	11.98	5.75	0.073	1.331	0.573	0.032
$CsPb_{0.125}Si_{0.375}(GeSn)_{0.5}Br_3$	47.45	9.81	9.00	0.023	1.169	4.934	0.016
$CsPb_{0.125}Ge_{0.375}(SiSn)_{0.5}Br_3$	47.74	11.66	8.37	0.038	1.165	1.655	0.019
$CsPb_{0.125}Sn_{0.375}(SiGe)_{0.5}Br_3$	47.79	12.31	8.26	0.040	1.202	1.376	0.019
$CsSi_{0.125}Ge_{0.375}(SnPb)_{0.5}I_3$	39.52	11.65	8.22	0.055	1.071	0.788	0.018
$CsSi_{0.125}Sn_{0.375}(GePb)_{0.5}I_3$	38.40	11.19	7.12	0.049	1.200	0.986	0.020
$CsSi_{0.125}Pb_{0.375}(GeSn)_{0.5}I_3$	37.74	11.64	6.42	0.064	1.316	0.600	0.021
$CsGe_{0.125}Si_{0.375}(SnPb)_{0.5}I_3$	39.70	11.51	8.94	0.034	1.157	1.668	0.014
$CsGe_{0.125}Sn_{0.375}(SiPb)_{0.5}I_3$	37.74	11.30	6.91	0.038	1.298	1.391	0.019
$CsGe_{0.125}Pb_{0.375}(SiSn)_{0.5}I_3$	37.15	9.99	6.76	0.058	1.339	0.930	0.018
$CsSn_{0.125}Si_{0.375}(GePb)_{0.5}I_3$	39.12	10.60	6.69	0.039	1.048	1.577	0.028
$CsSn_{0.125}Ge_{0.375}(SiPb)_{0.5}I_3$	39.66	11.69	7.98	0.049	1.015	0.934	0.021
$CsSn_{0.125}Pb_{0.375}(SiGe)_{0.5}I_3$	38.20	11.73	6.34	0.062	1.158	0.627	0.027
$CsPb_{0.125}Si_{0.375}(GeSn)_{0.5}I_3$	39.50	8.11	9.55	0.021	0.947	6.887	0.016
$CsPb_{0.125}Ge_{0.375}(SiSn)_{0.5}I_3$	39.17	10.24	8.87	0.028	0.959	2.782	0.018
$CsPb_{0.125}Sn_{0.375}(SiGe)_{0.5}I_3$	39.10	10.56	8.86	0.028	1.059	2.610	0.016

Table S4. The Seebeck coefficient S (μV/K), electric conductivity σ (10^5 $\Omega^{-1}m^{-1}$), power factor PF ($10^{-3}WK^{-2}m^{-1}$) and figure of merit ZT_{max}. Note: Symbol [a] represents the results from ref. [1].

	K				Cu				Ag			
	S	σ	PF	ZT_{max}	S	σ	PF	ZT_{max}	S	σ	PF	ZT_{max}
$APbBr_3$	246.85<br>[a]220.78	0.48<br>[a]0.60	2.93<br>[a]2.94	0.922<br>[a]0.845	32.38	29.74	3.12	0.042	37.24	27.47	3.81	0.055
$APbI_3$	265.36	0.37	2.61	1.104	44.45	33.55	6.63	0.080	59.09	14.69	5.13	0.137
$AB^{eq}Br_3$	278.94	0.69	5.34	1.225	21.92	47.25	2.27	0.019	14.40	86.72	1.80	0.008
$AB^{eq}I_3$	262.82	0.81	5.60	1.456	21.97	35.54	1.72	0.019	27.67	47.62	3.65	0.031
$ASi_{0.125}Ge_{0.375}(SnPb)_{0.5}Br_3$	269.30	0.67	4.83	1.133	27.40	34.77	2.61	0.030	21.47	46.29	2.13	0.018
$ASi_{0.125}Sn_{0.375}(GePb)_{0.5}Br_3$	266.21	0.73	5.20	1.191	8.03	58.16	0.37	0.003	7.75	63.80	0.38	0.002
$ASi_{0.125}Pb_{0.375}(GeSn)_{0.5}Br_3$	261.96	0.59	4.05	1.027	3.66	61.25	0.08	0.001	7.11	70.82	0.36	0.002
$ASi_{0.125}Ge_{0.375}(SnPb)_{0.5}I_3$	282.71	0.59	4.75	1.445	27.40	51.65	3.88	0.030	45.17	18.35	3.74	0.081
$ASi_{0.125}Sn_{0.375}(GePb)_{0.5}I_3$	241.47	1.01	5.90	1.379	66.04	25.71	11.21	0.173	14.86	56.73	1.25	0.009
$ASi_{0.125}Pb_{0.375}(GeSn)_{0.5}I_3$	286.17	0.48	3.96	1.345	38.71	27.14	4.07	0.060	23.18	63.54	3.41	0.022
$AGe_{0.125}Si_{0.375}(SnPb)_{0.5}Br_3$	272.76	0.92	6.83	1.372	14.24	56.21	1.14	0.008	10.69	59.17	0.68	0.005
$AGe_{0.125}Sn_{0.375}(SiPb)_{0.5}Br_3$	270.04	0.77	5.60	1.248	12.90	73.79	1.23	0.007	15.43	86.21	2.05	0.010
$AGe_{0.125}Pb_{0.375}(SiSn)_{0.5}Br_3$	277.03	0.65	5.01	1.209	11.70	55.75	0.76	0.005	7.39	70.65	0.39	0.002
$AGe_{0.125}Si_{0.375}(SnPb)_{0.5}I_3$	166.17	3.49	9.63	0.924	20.26	47.39	1.95	0.017	43.95	19.47	3.76	0.077
$AGe_{0.125}Sn_{0.375}(SiPb)_{0.5}I_3$	167.65	3.10	8.70	0.928	35.17	38.22	4.73	0.050	24.25	59.36	3.49	0.024
$AGe_{0.125}Pb_{0.375}(SiSn)_{0.5}I_3$	293.56	0.64	5.50	1.642	35.46	33.30	4.19	0.050	20.84	62.26	2.71	0.018
$ASn_{0.125}Si_{0.375}(GePb)_{0.5}Br_3$	284.09	0.92	7.44	1.472	15.54	68.07	1.64	0.010	6.71	62.26	0.28	0.002
$ASn_{0.125}Ge_{0.375}(SiPb)_{0.5}Br_3$	275.41	0.74	5.58	1.228	19.88	56.10	2.22	0.016	9.59	57.69	0.53	0.004
$ASn_{0.125}Pb_{0.375}(SiGe)_{0.5}Br_3$	260.50	0.67	4.53	1.071	23.43	30.10	1.65	0.022	32.61	36.52	3.88	0.042
$ASn_{0.125}Si_{0.375}(GePb)_{0.5}I_3$	317.41	0.70	7.10	1.944	18.94	61.34	2.20	0.014	22.93	54.25	2.85	0.021
$ASn_{0.125}Ge_{0.375}(SiPb)_{0.5}I_3$	289.14	0.62	5.21	1.523	26.30	55.87	3.86	0.028	25.68	70.32	4.64	0.027
$ASn_{0.125}Pb_{0.375}(SiGe)_{0.5}I_3$	278.35	0.53	4.14	1.325	37.89	26.84	3.85	0.057	22.81	48.34	2.52	0.021
$APb_{0.125}Si_{0.375}(GeSn)_{0.5}Br_3$	245.61<br>[a]372.72	31.98<br>[a]1.33	192.94<br>[a]18.61	2.383<br>[a]3.012	12.85	104.71	1.73	0.007	7.10	75.07	0.38	0.002
$APb_{0.125}Ge_{0.375}(SiSn)_{0.5}Br_3$	299.96	0.68	6.11	1.355	19.32	66.47	2.48	0.015	13.11	99.81	1.72	0.007

$APb_{0.125}Sn_{0.375}(SiGe)_{0.5}Br_3$	250.19	1.27	7.93	1.351	12.46	81.05	1.26	0.006	12.20	74.45	1.11	0.006
$APb_{0.125}Si_{0.375}(GeSn)_{0.5}I_3$	159.57	0.56	1.42	0.425	26.39	75.78	5.28	0.028	24.42	52.55	3.13	0.024
$APb_{0.125}Ge_{0.375}(SiSn)_{0.5}I_3$	202.30	6.87	28.10	1.503	27.89	46.24	3.60	0.031	23.23	69.63	3.76	0.022
$APb_{0.125}Sn_{0.375}(SiGe)_{0.5}I_3$	184.71	6.77	23.09	1.253	43.30	19.46	3.65	0.074	23.84	53.41	3.03	0.023

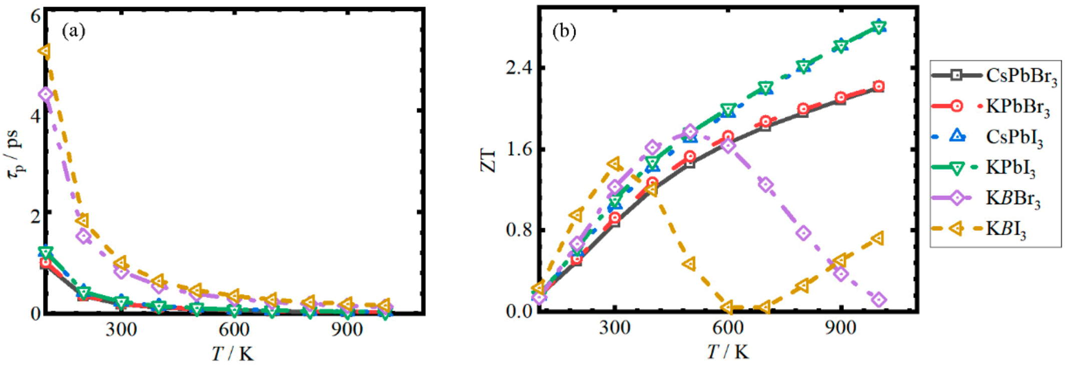

Figure S4. (**a**) The relaxation time τ_p and (**b**) ZT values of $APbX_3$ (A=Cs, K) and $KSi_{0.25}Ge_{0.25}Sn_{0.25}Pb_{0.25}X_3$ MHPs as a function of temperature, respectively.

References

1. W. Lee, H. Li, A.B. Wong, D. Zhang, M. Lai, Y. Yu, Q. Kong, E. Lin, J.J. Urban, J.C.J.P.o.t.N.A.o.S. Grossman, Ultralow thermal conductivity in all-inorganic halide perovskites, 114(33) (2017) 8693-8697.
